# Activation of p38 Mitogen-Activated Protein Kinase in Gaucher’s Disease

**DOI:** 10.1371/journal.pone.0136633

**Published:** 2015-08-27

**Authors:** Kazuyuki Kitatani, Masayuki Wada, David Perry, Toshinori Usui, Ying Sun, Lina M. Obeid, Nobuo Yaegashi, Gregory A. Grabowski, Yusuf A. Hannun

**Affiliations:** 1 Tohoku Medical Megabank Organization, Tohoku University, Sendai, Japan; 2 Department of Obstetrics and Gynecology, Tohoku University Graduate School of Medicine, Sendai, Japan; 3 Stony Brook Cancer Center, Stony Brook University, Stony Brook, New York, United States of America; 4 Department of Medicine, Medical University of South Carolina, Charleston, South Carolina, United States of America; 5 Division of Human Genetics, Cincinnati Children's Hospital Medical Center, Cincinnati, Ohio, United States of America; 6 Departments of Pediatrics, University of Cincinnati College of Medicine, Cincinnati, Ohio, United States of America; 7 Northport Veterans Affairs Hospital, Northport, New York, United States of America; 8 Department of Medicine, Stony Brook University, Stony Brook, New York, United States of America; Dasman Diabetes Institute, KUWAIT

## Abstract

Gaucher’s disease is caused by defects in acid β-glucosidase 1 (GBA1) and has been also proposed as an inflammatory disease. GBA1 cleaves glucosylceramide to form ceramide, an established bioactive lipid, and defects in GBA1 lead to aberrant accumulation in glucosylceramide and insufficient formation of ceramide. We investigated if the pro-inflammatory kinase p38 is activated in Gaucher’s disease, since ceramide has been proposed to suppress p38 activation. Three Gaucher’s disease mouse models were employed, and p38 was found to be activated in lung and liver tissues of all Gaucher’s disease mice. Most interestingly, neuronopathic Gaucher’s disease type mice, but not non-neuronopathic ones, displayed significant activation of p38 and up-regulation of p38-inducible proinflammatory cytokines in brain tissues. In addition, all type of Gaucher’s disease mice also showed increases in serum IL-6. As cellular signalling is believed to represent an *in vivo* inflammatory phenotype in Gaucher’s disease, activation of p38 and possibly its-associated formation of proinflammatory cytokines were assessed in fibroblasts established from neuronopathic Gaucher’s disease mice. In mouse Gaucher’s disease cells, p38 activation and IL-6 formation by TNF-α treatment were enhanced as compared to those of wild type. Furthermore, human fibroblasts from Gaucher’s disease patients also displayed increases in p38 activation and IL-6 formation as comparison to healthy counterpart. These results raise the potential that proinflammatory responses such as p38 activation and IL-6 formation are augmented in Gaucher’s disease.

## Introduction

Gaucher’s disease is the most common lysosomal storage disorder caused by mutations in a gene encoding a lysosomal enzyme, acid β-glucosidase 1 (GBA1) [1–5]. The enzyme degrades glucosylceramide to form ceramide, and the defects in the catalytic activity in Gaucher’s disease result in intracellular accumulation of undegraded substrates (glucosylceramide and glucosylsphngosine). The unifying primary clinical manifestations in Gaucher’s disease include hepatosplenomegaly, hematologic abnormalities, anemia, thrombocytopenia, and bony lesions [6]. The disease has classically been divided into three major subtypes, namely types I, II, and III, which are distinguished by the presence of or absence of neuronopathic manifestations [7].

The classical hallmark of the disease is the presence of the Gaucher cell, a macrophage containing much of the stored glucosylceramide found in tissues, which is believed to cause the clinical manifestations of the disease. However, the pathogenesis of Gaucher’s disease appears to extend beyond simple accumulation of glucosylceramide in Gaucher cells and seems to involve systemic inflammatory responses [8, 9]. Importantly, Gaucher’s disease patients have been reported to display elevated serum concentrations of hematopoietic growth factors and proinflammatory cytokines, including monocyte/macrophage colony-stimulating factor, TNF-α, interleukin (IL)-1β, IL-8, and IL-6 [10–12]. In addition, Mizukami *et al*. also demonstrated that Gaucher’s disease is linked to systemic inflammation in a mouse model [13]. Interestingly, brain inflammation is likely to contribute to neuronal cells death in neuronpathic Gaucher’s disease mice [8]. In fact, neurodegenerative diseases are associated with brain inflammation, and preclinical studies demonstrated that sphingolipidoses such as Sandhoff disease [14] and Niemann-Pick disease [15] benefited from anti-inflammatory therapy. Collectively, inflammation is suggested to play a role in the pathogenesis of Gaucher’s disease.

Our previous studies suggested that GBA1 participates in a pathway that leads to the formation of ceramide which in turn serves as an inhibitory lipid in activation of the proinflammatory kinase p38 [16, 17], raising the potential of ceramide as an anti-inflammatory lipid. Thus, insufficient formation of ceramide caused by GBA1 defects was assumed to promote p38 activation and its-driven inflammatory responses in Gaucher’s disease.

In preclinical and clinical studies, p38 is a therapeutic target in inflammatory disease such as rheumatoid arthritis, Crohn’s disease, and neurodegenerative diseases [18–20]. In the present studies, we assessed if p38 is activated in Gaucher’s disease *in vivo* and *in vitro*. Those studies provide insight for potential anti-inflammatory therapy targeting p38 for Gaucher’s disease.

## Materials and Methods

### Antibodies and reagents

Phospho/active p38 antibody (V1211) was purchased from Promega (Madison, WI). Horseradish-peroxidase-conjugated antibodies for mouse (sc2005) and rabbit (sc2004) IgG were from Santa Cruz Biotechnology. A mouse monoclonal antibody specific for β-actin (A5441) was from Sigma. Recombinant human tumor necrosis factor-α (TNF-α, 300-01A) was from Peprotech. Halt Phosphatase Inhibitor Cocktail was from Pierce. Phorbol 12-myristate 13-acetate (PMA, 524400) was from Calbiochem. SuperSignal West Dura Extended Duration Substrate was from Thermo Scientific.

Human fibroblasts from healthy (wild type GBA1: catalog number AG14580) and Gaucher’s disease patients (case-1, catalog number GM10915; case-2, catalog number GM00877) were purchased from the Coriell Institute For Medical Research. The case-1 patient harboring homozygous L444P mutation (expired at age 7.5) was classified into type 1 according to the profile of the Coriell Institute For Medical Research and has clinical manifestations including severe hepatosplenomegaly, bilateral sensorineural hearing loss, but no other CNS findings. The case-2 patient also harboring homozygous L444P mutation (expired at age 1) was classified into type 2 with clinical manifestations including hepatosplenomegaly, strabismus, and trismus.

### Ethics statement

The use of murine models and experimental protocols was reviewed and approved by the institutional animal care and use committee at Cincinnati Children's Hospital Medical Center (Cincinnati, OH, USA) prior to the start of this study and. The ID number is 2D12113.

### Gaucher’s disease mice

Three types of Gaucher’s disease mouse models including V394L/V394L (V394L) [21], D409H/D409H (D409H) [21], and V394L/PS-NA [22] were maintained in a microisolator pathogen-free environment. Mice (10 weeks, female) were euthanized with CO_2_ suffocation followed by cervical dislocation and then tissues and serum were collected.

### Cell culture

Human fibroblasts established from patients with Gaucher’s disease and healthy volunteer were grown in α-MEM medium supplemented with 10–15% fetal bovine serum (FBS). Mouse fibroblasts established from wild type or Gaucher’s disease model mouse were grown in DMEM medium supplemented with 10% FBS. Cells were maintained at <80% confluence under standard incubator conditions (humidified atmosphere, 95% air, 5% CO_2_, 37°C).

### IL-6 ELISA assay

For measuring mouse serum IL-6, blood samples were allowed to clot for 2 h at room temperature. The clotted materials were removed by centrifugation at 1,000 × g for 10 min, and then the supernatant serum was collected. IL-6 production in cell culture supernatants was normalized to proteins extracted from adherent cells. Levels of IL-6 in culture supernatants or mouse serum were measured using commercially available IL-6 ELISA (Quantikine; R&D Systems).

### Immunoblotting

Tissues from mice (10 weeks of age, female) were homogenized in M-PER (Mammalian Protein Extraction Reagent), and then proteins were extracted as described [22]. For human or mouse fibroblast studies, cultured cells were washed three times with phosphate-buffered saline (PBS) supplemented with Halt Phosphatase Inhibitor Cocktail and lysed using Laemmli buffer. Protein concentrations were determined using the BCA method [23]. The protein samples (20 μg) were subjected to SDS-PAGE (4–20% gradient gels). Proteins were electrophoretically transferred to nitrocellulose membranes, blocked with PBS/0.1% Tween 20 (PBS-T) containing 5% nonfat dried milk, washed with PBS-T, and incubated with antibodies for β-actin or phospho–p38 in PBS-T containing 5% nonfat dried milk. The blots were washed with PBS-T and then incubated with a secondary antibody conjugated with horseradish peroxidase in PBS-T containing 5% nonfat dried milk. Detection was performed using enhanced chemiluminescence reagent, and quantification of the chemiluminescent signals was performed with a digital imaging system (VersaDoc, Bio-Rad).

### Quantitative real-time PCR (Q-RT-PCR)

RNAs in mouse tissues were extracted using TOTALLY RNA kit (Ambion Inc., catalog number AM1910). Reverse transcription of total RNA (1 μg) was carried out using RT^2^ first strand kit (QIAGEN, catalog number 330401). The resulting cDNAs were used in the quantitative real-time PCR to determine the mRNA levels. Real-time PCR was performed on an iCycler iQ Multicolor Real-Time PCR Detection System (Bio-Rad Laboratories, Hercules, CA, USA) with iQ SYBRE Green Supermix (Bio-Rad). The mouse β-actin gene was used as an internal reference control to normalize relative levels of gene expression, and expression of each transcript is presented as arbitrary units (AU). The following primers were used: β-actin (forward, TCCTCCCTGGAGAAGAGCTA; reverse, CCAGACAGCACTGTGTTGGC); IL-6 (forward, TCCAGTTGCCTTCTTGGGAC; reverse, GTGTAATTAAGCCTCCGACTTG), 4331182; p38α (forward, CAGTTCAACTCCATGCCATC; reverse, CCCTAACACAGCATGGTCAC); p38β (forward, CAGTCCTGAAGTTCTGGCAA; reverse, TGGAAGACACTGCTGAGGTC); p38γ (forward, GGCCCAGAAATATGACGACT; reverse, TCTCCTTTGGAACTCTGGCT); p38δ (forward, TCGACATCTGGTCTGTTGGT; reverse, GGTCAGCTGGTCCAGGTAGT). TNF-α (forward, AATGGCCTCCCTCTCATCAGTT; reverse, CCACTTGGTGGTTTGCTACGA).

### Statistical analysis

Results are presented as means ± SEM. Statistical analyses were performed using GraphPad Prism and Instat. Comparison between two groups was carried out by unpaired Student’s *t* test.

## Results

### p38 is activated in Gaucher’s disease mice

**Fig 1:**
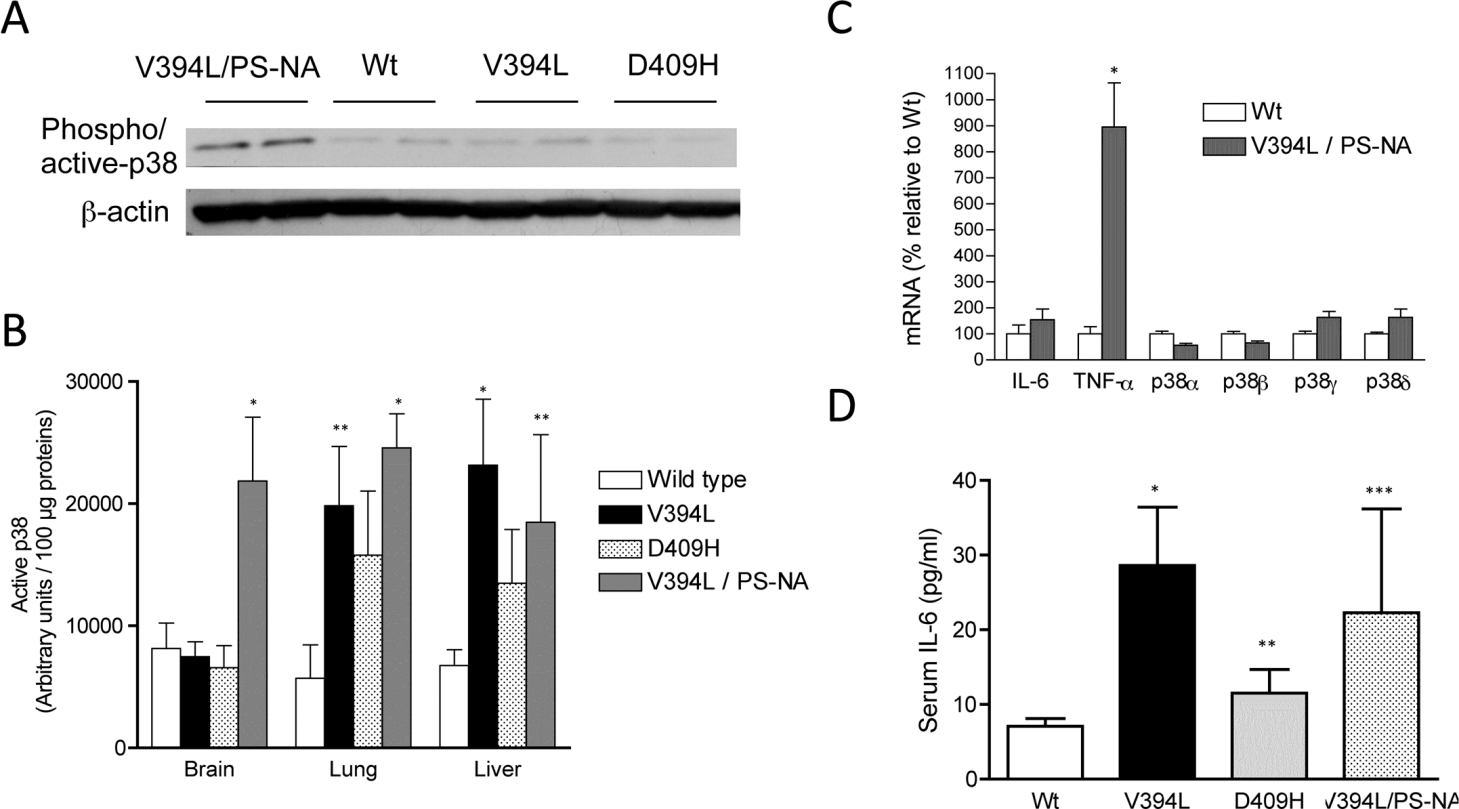
p38 activation and IL-6 formation in a Gaucher’s disease mouse model. Proteins were extracted from tissues (brain, lung, and liver) of wild type mice or Gaucher disease mouse models (V394L, D409H, and V394L/PS-NA) and then subjected to immunoblot analysis with antibodies specific for phospho- p38 and β-actin. Equal amounts of protein were loaded in each lane, and the representative results of brain tissues are shown (**A**). Amounts of active/phospho-p38 were estimated by measuring the density of bands of phospho-p38 and expressed as arbitrary units (**B**). The data represent mean ± S.E. (n = 4–7). *, *p* < 0.02; **, *p* < 0.05. (**C**) mRNA was extracted from brain tissues from wild type and neuropathic Gaucher disease model V394L/PS-NA mice, and mRNAs of p38 isoforms and IL-6 were determined by the quantitative real time PCR. The data represent mean ± S.E. (n = 5). TNF-α, *p* < 0.0006 (*). (**D**) Serum IL-6 levels from wild type mice or Gaucher disease mouse models (V394L, D409H, and V394L/PS-NA) were determined by the ELISA system. The data represent mean ± S.E. Wild type, n = 21; V394L, n = 10; D409H, n = 7; V394L/PS-NA, n = 7. Wt *vs* V394L, *p* < 0.0006 (*); Wt *vs* D409, *p* < 0.03 (**); Wt *vs* V394L/PS-NA, *p* < 0.05 (***).

To evaluate p38 activation in Gaucher’s disease *in vivo*, three types of Gaucher’s disease mouse models were employed, including V394L, D409H, and V394L/PS-NA. The former two types have been established as homozygous for *GBA1* point mutant knock-in mice with minor glucosylceramide accumulation in viscera and without significant phenotypes such as neuro-degeneration and shorter life span [21]. The last has been generated by crossing V394L homozygous with the mouse with hypomorphic expression of the prosaposin transgenes (5–45% of wild type, PS-NA), showing glucosylceramide accumulation in multiple organs, several phenotypes including neuro-degeneration and shorter life span [22]. Phospho/active p38 in tissues was determined by immunoblotting. p38 in the brain tissues of neuronopathic mice (V394L/PS-NA) was activated as compared with those of wild type, whereas other Gaucher’s disease mice (V394L and D409H) did not show significant activation of p38 (Fig 1A and 1B). p38 activation in brain tissues is correlated with neuronopathic phenotype. As to other tissues including liver and lung, the levels of active p38 in all types of Gaucher’s disease mice were increased as compared with those of wild type (Fig 1B).

Proinflammatory cytokines in brain have pathological roles in neurodegenerative central nervous system. Campbell *et al*. have reported that cerebral overexpression of IL-6 induced neurologic disease [24]. mRNA levels of the major proinflammatory cytokine TNF-α were highly elevated in brain tissues of neuronopathic V394L/PS-NA mice relative to those of wild type mice (Fig 1C). IL-6 mRNA increases were not statistically significant (Fig 1C); however, its mRNA levels were prone to increase in brain tissues of neurodegenerative V394L/PS-NA. Therefore, neuropathic phenotype is believed to associate with the elevation of inflammatory cytokines.

Activation of p38 is involved in forming a myriad of inflammatory mediators, and p38 is a predominant kinase responsible for generating proinflammatory cytokines such as TNF-α and IL-6 [18]. In fact, previous studies showed that *GBA1* knockdown potentiates p38-dependent formation of IL-6 in human cell lines [16], and IL-6 has been shown to increase in serum of patients with Gaucher’s disease [11, 12]. We determined serum IL-6 in Gaucher’s disease model mice using an ELISA system (Fig 1D). Serum IL-6 in the mice (V394L, D409L, and V394L/PS-NA) was 28.5 ± 7.8, 12.8 ± 2.7, and 23.1 ± 13.6 pg/ml, respectively. All values were significantly higher as compared with that of wild type (7.1 ± 1.0 pg/ml). Those results show that serum IL-6 is increased with GBA1 defects. This elevation is consistent with that of patients with Gaucher’s disease [11].

### p38 is activated in cellular proinflammatory responses in mouse Gaucher’s disease fibroblasts

**Fig 2:**
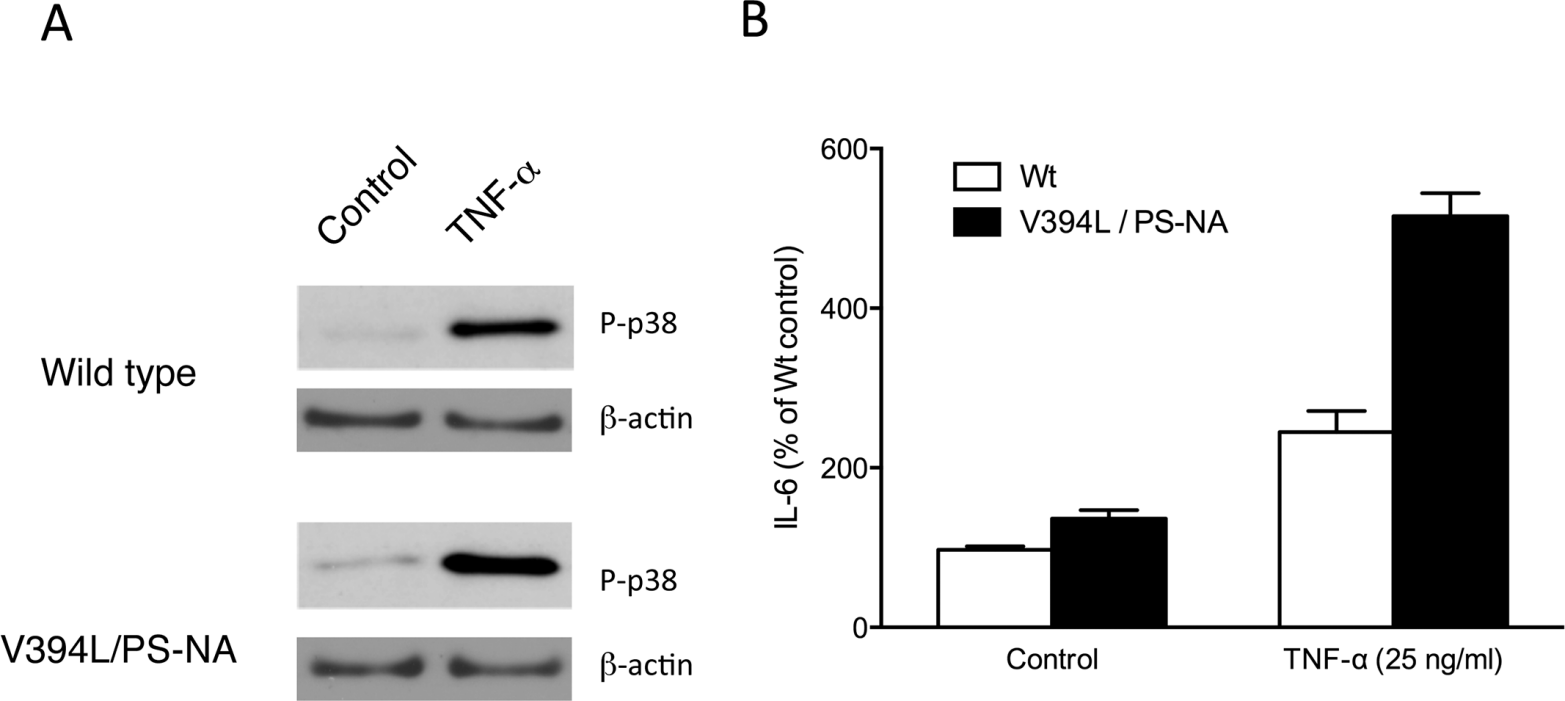
p38 activation and IL-6 formation in Gaucher’s disease mouse fibroblasts. **A.** Fibroblasts from wild type mouse or V394L/PS-NA mouse were stimulated with 25 ng/ml TNF-α for 30 min, and then proteins were extracted. Proteins were subjected to immunoblot analysis with antibodies specific for phospho-p38 (p-p38) and β-actin. Equal amounts of protein were loaded in each lane, and the representative results are shown. **B.** After 6 h stimulation, levels of IL-6 in culture supernatants were measured using ELISA system. The data represent mean ± S.E. (n = 3).

The *in vivo* studies above raised the possibility of a pathological role for p38 activation in Gaucher disease. p38 is known to serve as a proinflammatory kinase that is activated in response to proinflammatory cytokines or inflammation inducers such as PMA [25]. To determine if p38 is activated in proinflammatory responses of fibroblast cells derived from Gaucher’s disease mouse (V394L/PS-NA), those cells were stimulated with TNF-α, known as a potent inflammatory cytokine. Both basal and TNF-α-treated fibroblasts established from V394L/PS-NA mouse displayed higher p38 activation as compared to those of wild type, respectively (Fig 2A). Consistent with potentiated activation of p38, IL-6 formation was also facilitated in mouse Gaucher’s disease fibroblasts (Fig 2B).

### Gaucher’s disease patients-derived fibroblasts show increased p38 activation and IL-6 formation

**Fig 3:**
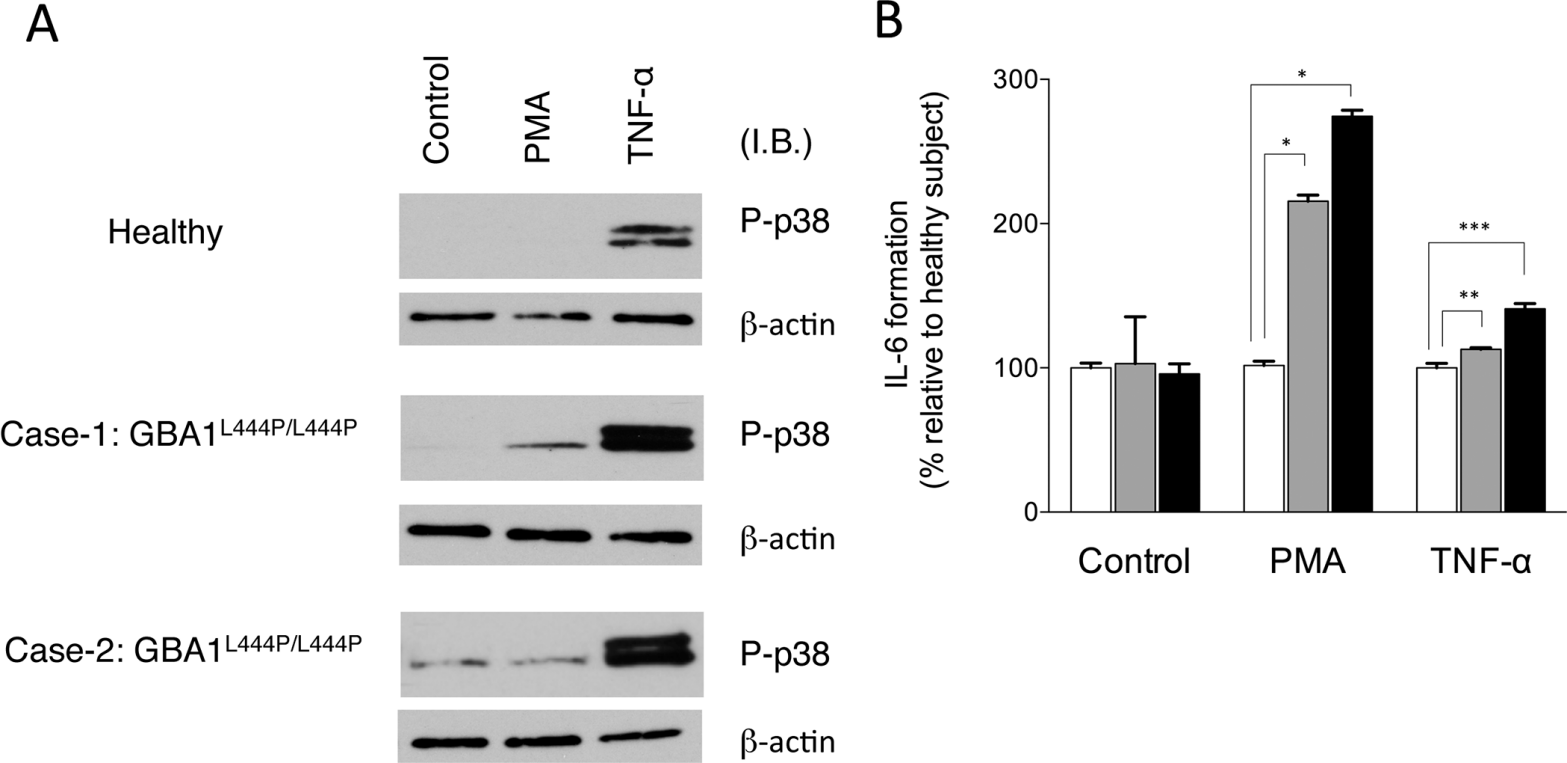
p38 activation and IL-6 formation in fibroblasts from Gaucher’s disease patients harboring GBA1^L444P/L444P^. **A.** Human fibroblasts were stimulated with 100 nM PMA or 25 ng/ml TNF-α for 30 min. Proteins were subjected to immunoblot analysis with antibodies specific for phospho/active-p38 (p-p38) and β-actin. Equal amounts of protein were loaded in each lane, and the representative results are shown. **B.** After 6 h stimulation, levels of IL-6 in culture supernatants were measured using ELISA system. IL-6 values in control, PMA and TNF-α are expressed as percentage relative to those of healthy subject, respectively. Data represent mean ± S.E. (n = 3). *, *p* < 0.001; **, *p* < 0.02; ***, *p* < 0.002.

Genetic approaches and pharmacological approaches demonstrated that GBA1 defects facilitate p38 activation *in vitro* [16] and *in vivo* (above); however, whether p38 is highly activated in human Gaucher’s disease is not known. Human fibroblasts established from Gaucher’s disease patients harboring homozygous L444P were employed to assess effects of Gaucher’s disease on p38 activation and IL-6 formation. Those cells were stimulated with PMA or TNF-α, and then p38 activation was determined by immunoblotting. p38 was activated in response to TNF-α treatment in fibroblasts from healthy, whereas fibroblasts from both two Gaucher’s patients displayed significant increases in p38 activation in response to TNF-α as compared to that of healthy (Fig 3A). Similar to mouse fibroblasts, human Gaucher’s fibroblasts also displayed increased formation of IL-6 in response to individual treatments with PMA and TNF-α (Fig 3B).

## Discussion

The results from this study provide significant evidence to support the emergence of Gaucher’s disease as an inflammatory disease. Our studies ([16] and the current results) suggest that the p38 pro-inflammatory kinase is activated in Gaucher’s disease. Especially, p38 activation in brain tissues is associated with the neurodegeneration phenotype in Gaucher’s disease. Therefore, p38 activation is proposed to play a role in the inflammation-associated pathogenesis in Gaucher’s disease.

In brain tissues, p38 activation was specific to neuronopathic Gaucher’s disease V394L/PS-NA mice (Fig 1A and 1B). Several types of neuronopathic Gaucher’s disease mouse models have been established by genetic engineering. Neuronopathic Gaucher’s disease mouse model (GBA1^V394L/V394L^, saposin C^-/-^) has been also generated, displaying shortened life span and brain proinflammation involving elevations in active p38, IL-6 and TNF-α mRNAs [26]. Importantly, *in vivo* treatment with a pharmacological chaperon, isofagomine, known to enhance GBA1 function in Gaucher’s disease fibroblasts, suppressed the increased p38 activation and TNF-α formation in brain tissues, and extended life span [26]. Therefore, GBA1 defects are suggested to facilitate p38 that serves as a proinflammatory kinase in brain tissues.

p38 MAPK is classified into four isoforms including p38α, p38β, p38γ, and p38δ [27, 28]. GBA1-regulated ceramide in cancer cell lines is suggested to down-regulate activation of p38δ by activating ceramide-activated protein phosphatases [16, 17]. Which p38 isoform is predominantly activated in Gaucher’s disease remains to be determined, although mRNA expression of all p38 isoforms was detected in mouse brain tissues (Fig 1C).

p38 has been implicated in cellular inflammatory responses [29], differentiation [30] and cell death [30], and is believed to serve as a key kinase that mediates the production of inflammatory mediators such as proinflammatory cytokines, prostaglandins and reactive oxygen species. Importantly, p38 serves as a crucial molecule that promotes the formation of IL-6 and TNF-α, *in vivo* and *in vitro*. Experimentally, overexpression of those cytokines in the central nervous system of transgenic mice triggers central nervous system inflammation and degeneration [24, 31–33]. Therefore, p38 activation and/or increased formation of these cytokines might play roles in the pathogenesis of Gaucher’s disease.

In summary, our studies demonstrate that p38 signaling is activated in Gaucher disease and raise the possibility that p38-driven inflammation is involved in the disease pathogenesis.

## References

[1] Brady RO. (1978) Sphingolipidoses, Annu Rev Biochem. 47, 687–713. 98102 10.1146/annurev.bi.47.070178.003351

[2] Dinur T, Osiecki KM, Legler G, Gatt S, Desnick RJ, Grabowski GA (1986) Human acid beta-glucosidase: isolation and amino acid sequence of a peptide containing the catalytic site, Proceedings of the National Academy of Sciences of the United States of America. 83, 1660–4. 3456607 10.1073/pnas.83.6.1660 PMC323143

[3] Grabowski GA (1993) Gaucher disease. Enzymology, genetics, and treatment, Adv Hum Genet. 21, 377–441. 8317294

[4] Futerman AH, van Meer G (2004) The cell biology of lysosomal storage disorders, Nat Rev Mol Cell Biol. 5, 554–65. 15232573 10.1038/nrm1423

[5] Mistry PK, Cox TM (1993) The glucocerebrosidase locus in Gaucher's disease: molecular analysis of a lysosomal enzyme, Journal of medical genetics. 30, 889–94. 8301642 10.1136/jmg.30.11.889 PMC1016594

[6] Grabowski GA, Hopkin RJ (2003) Enzyme therapy for lysosomal storage disease: principles, practice, and prospects, Annual review of genomics and human genetics. 4, 403–36. 10.1146/annurev.genom.4.070802.110415 14527307

[7] Jmoudiak M, Futerman AH (2005) Gaucher disease: pathological mechanisms and modern management, British journal of haematology. 129, 178–88. 15813845 10.1111/j.1365-2141.2004.05351.x

[8] Vitner EB, Farfel-Becker T, Eilam R, Biton I, Futerman AH (2012) Contribution of brain inflammation to neuronal cell death in neuronopathic forms of Gaucher's disease, Brain. 135, 1724–35. 22566609 10.1093/brain/aws095

[9] Pandey MK, Rani R., Zhang W, Setchell K, Grabowski GA (2012) Immunological cell type characterization and Th1-Th17 cytokine production in a mouse model of Gaucher disease, Molecular genetics and metabolism. 106, 310–22. 22595426 10.1016/j.ymgme.2012.04.020 PMC3382074

[10] Hollak CE, Evers L, Aerts JM, van Oers MH (1997) Elevated levels of M-CSF, sCD14 and IL8 in type 1 Gaucher disease, Blood Cells Mol Dis. 23, 201–12. 9236158 10.1006/bcmd.1997.0137

[11] Barak V, Acker M, Nisman B, Kalickman I, Abrahamov A, Zimran A, Yatziv S. (1999) Cytokines in Gaucher's disease, Eur Cytokine Netw. 10, 205–10. 10400826

[12] Allen MJ, Myer BJ, Khokher AM, Rushton N, Cox TM (1997) Pro-inflammatory cytokines and the pathogenesis of Gaucher's disease: increased release of interleukin-6 and interleukin-10, QJM. 90, 19–25. 9093585 10.1093/qjmed/90.1.19

[13] Mizukami H, Mi Y, Wada R, Kono M, Yamashita T, Liu Y, et al (2002) Systemic inflammation in glucocerebrosidase-deficient mice with minimal glucosylceramide storage, J Clin Invest. 109, 1215–21. 11994410 10.1172/JCI14530 PMC150961

[14] Jeyakumar M, Smith DA, Williams IM, Borja MC, Neville DC, Butters TD, et al (2004) NSAIDs increase survival in the Sandhoff disease mouse: synergy with N-butyldeoxynojirimycin, Annals of neurology. 56, 642–9. 15505823 10.1002/ana.20242

[15] Smith D, Wallom KL, Williams IM, Jeyakumar M, Platt FM (2009) Beneficial effects of anti-inflammatory therapy in a mouse model of Niemann-Pick disease type C1, Neurobiology of disease. 36, 242–51. 19632328 10.1016/j.nbd.2009.07.010

[16] Kitatani K, Sheldon K, Anelli V, Jenkins RW, Sun Y, Grabowski GA, et al (2009) Acid beta-glucosidase 1 counteracts p38delta-dependent induction of interleukin-6: possible role for ceramide as an anti-inflammatory lipid, The Journal of biological chemistry. 284, 12979–88. 19279008 10.1074/jbc.M809500200 PMC2676030

[17] Kitatani K, Sheldon K, Rajagopalan V, Anelli V, Jenkins RW, Sun Y, et al (2009) Involvement of acid beta-glucosidase 1 in the salvage pathway of ceramide formation, The Journal of biological chemistry. 284, 12972–8. 19279011 10.1074/jbc.M802790200 PMC2676029

[18] Cuenda A, Rousseau S (2007) p38 MAP-kinases pathway regulation, function and role in human diseases, Biochimica et biophysica acta. 1773, 1358–75. 17481747 10.1016/j.bbamcr.2007.03.010

[19] Coulthard LR, White DE, Jones DL, McDermott MF, Burchill SA (2009) p38(MAPK): stress responses from molecular mechanisms to therapeutics, Trends in molecular medicine. 15, 369–79. 19665431 10.1016/j.molmed.2009.06.005 PMC3016890

[20] Bachstetter AD, Van Eldik LJ (2010) The p38 MAP Kinase Family as Regulators of Proinflammatory Cytokine Production in Degenerative Diseases of the CNS, Aging and disease. 1, 199–211. 22720195 PMC3377763

[21] Xu YH, Quinn B, Witte D, Grabowski GA (2003) Viable mouse models of acid beta-glucosidase deficiency: the defect in Gaucher disease, Am J Pathol. 163, 2093–101. 14578207 10.1016/s0002-9440(10)63566-3 PMC1892407

[22] Sun Y, Quinn B, Witte DP, Grabowski GA (2005) Gaucher disease mouse models: point mutations at the acid beta-glucosidase locus combined with low-level prosaposin expression lead to disease variants, J Lipid Res. 46, 2102–13. 16061944 10.1194/jlr.M500202-JLR200

[23] Smith PK, Krohn RI, Hermanson GT, Mallia AK, Gartner FH, Provenzano MD, et al (1985) Measurement of protein using bicinchoninic acid, Analytical biochemistry. 150, 76–85. 3843705 10.1016/0003-2697(85)90442-7

[24] Campbell IL, Abraham CR, Masliah E, Kemper P, Inglis JD, Oldstone MB, et al (1993) Neurologic disease induced in transgenic mice by cerebral overexpression of interleukin 6, Proceedings of the National Academy of Sciences of the United States of America. 90, 10061–5. 7694279 10.1073/pnas.90.21.10061 PMC47713

[25] Kitatani K, Idkowiak-Baldys J, Bielawski J, Taha TA, Jenkins RW, Senkal CE, et al (2006) Protein kinase C-induced activation of a ceramide/protein phosphatase 1 pathway leading to dephosphorylation of p38 MAPK, The Journal of biological chemistry. 281, 36793–802. 17030510 10.1074/jbc.M608137200

[26] Sun Y, Ran H, Liou B, Quinn B, Zamzow M, Zhang W, et al (2011) Isofagomine in vivo effects in a neuronopathic Gaucher disease mouse, PLoS One. 6, e19037. 21533102 10.1371/journal.pone.0019037 PMC3080394

[27] Kumar S, Boehm J, Lee JC (2003) p38 MAP kinases: key signalling molecules as therapeutic targets for inflammatory diseases, Nat Rev Drug Discov. 2, 717–26. 12951578 10.1038/nrd1177

[28] Cuadrado A, Nebreda AR (2010) Mechanisms and functions of p38 MAPK signalling, The Biochemical journal. 429, 403–17. 20626350 10.1042/BJ20100323

[29] Dean JL, Sully G, Clark AR, Saklatvala J (2004) The involvement of AU-rich element-binding proteins in p38 mitogen-activated protein kinase pathway-mediated mRNA stabilisation, Cellular signalling. 16, 1113–21. 15240006 10.1016/j.cellsig.2004.04.006

[30] Olson JM, Hallahan AR (2004) p38 MAP kinase: a convergence point in cancer therapy, Trends in molecular medicine. 10, 125–9. 15102355 10.1016/j.molmed.2004.01.007

[31] Probert L, Akassoglou K, Kassiotis G, Pasparakis M, Alexopoulou L, Kollias G. (1997) TNF-alpha transgenic and knockout models of CNS inflammation and degeneration, Journal of neuroimmunology. 72, 137–41. 9042105 10.1016/s0165-5728(96)00184-1

[32] Probert L, Akassoglou K, Pasparakis M, Kontogeorgos G, Kollias G (1995) Spontaneous inflammatory demyelinating disease in transgenic mice showing central nervous system-specific expression of tumor necrosis factor alpha, Proceedings of the National Academy of Sciences of the United States of America. 92, 11294–8. 7479982 10.1073/pnas.92.24.11294 PMC40618

[33] Munoz-Fernandez MA, Fresno M (1998) The role of tumour necrosis factor, interleukin 6, interferon-gamma and inducible nitric oxide synthase in the development and pathology of the nervous system, Progress in neurobiology. 56, 307–40. 9770242 10.1016/s0301-0082(98)00045-8

